# Bruxism and Temporomandibular Disorders: Current Advances and Future Challenges

**DOI:** 10.3390/jcm15145428

**Published:** 2026-07-10

**Authors:** Grzegorz Zieliński, Eiji Tanaka

**Affiliations:** 1 Department of Stomatognathic System Rehabilitation, Medical University of Lublin, 20-093 Lublin, Poland; 2 Department of Orthodontics and Dentofacial Orthopedics, Graduate School of Biomedical Sciences, Tokushima University, Tokushima 770-8504, Japan; etanaka@tokushima-u.ac.jp

Bruxism and temporomandibular disorders (TMD) are among the most prevalent disorders affecting the masticatory system [1,2,3]. The prevalence of sleep bruxism is estimated at approximately 21%, while awake bruxism affects around 23% of the population, and TMD is reported to affect as many as 34% of individuals. The global co-occurrence of bruxism and TMD was 17%. The mean prevalence of TMD among patients with bruxism was 63.5% [1]. An increasing body of evidence confirms the multifactorial aetiology of these disorders, involving biological, psychological, and social factors [2,4,5]. Therefore, diagnosis and treatment require an interdisciplinary approach integrating dental, medical, psychological, and rehabilitative knowledge [6,7].

The articles published in this Special Issue present a broad spectrum of contemporary research directions. The significant role of psychological factors in the pathogenesis of painful forms of TMD was confirmed in a study analysing the relationship between masticatory muscle thickness and psychological status in female patients with myogenous TMD (Contribution 1). The importance of the biopsychosocial model is also emphasised in a review addressing predictors of pain chronicity (Contribution 2) and in a paediatric population study demonstrating associations between TMD, sleep disturbances, and emotional problems [8] (Contribution 3).

Many publications included in this Special Issue focus on occlusal and parafunctional factors. Associations between skeletal class, musculoskeletal pain patterns, and TMD were analysed by Nowak et al. (Contribution 4), whereas the influence of parafunctions, such as tooth clenching and abnormal mandibular positioning, was assessed among medical students (Contribution 5). Additional data are provided by studies investigating gum chewing (Contribution 6), occlusal interferences (Contribution 7), and non-functional occlusal habits in patients with craniofacial deformities (Contribution 8).

A substantial part of this Special Issue is dedicated to modern diagnostic methods. The potential of surface electromyography in TMD diagnostics is presented by Zieliński and Ginszt (Contribution 9), while the application of ultrasonography and elastography of the masseter muscle is demonstrated in studies by Wójcicki et al. (Contribution 1) and Sasajima et al. (Contribution 10). Valuable insights are also provided by MRI analyses of the temporomandibular joint (TMJ) [9] (Contribution 11).

Another important area of research concerned sleep bruxism. Two studies employing polysomnography and electromyography provided detailed characterisation of this phenomenon in young adults (Contribution 12 and 13). The pathophysiological perspective was further expanded by reviews addressing the association between bruxism and sleep-related breathing disorders as well as genetic determinants (Contribution 14), together with the effects of exposure to hypobaric hypoxia (Contribution 15).

Therapeutic strategies also represent an important research area. Promising results have been reported for myofunctional therapy in myogenous TMD [10] (Contribution 16). The effectiveness of minimally invasive treatment approaches was assessed in a study investigating arthrocentesis with the use of hyaluronic acid and platelet-rich plasma (Contribution 17), as well as in reviews focusing on autologous injections (Contribution 18), arthroscopic discopexy techniques (Contribution 19), and the safety of intra-articular injections (Contribution 20). In cases of advanced disorders, outcomes following total TMJ arthroplasty were analysed [11] (Contribution 21). Further evidence supporting the need for an individualised and multidisciplinary therapeutic strategy was provided by Dąbkowska et al. (Contribution 22).

Attention should also be drawn to reports concerning oxidative stress biomarkers (Contribution 23), sex-related differences in the diagnosis of limited mouth opening (Contribution 24), the co-occurrence of TMD and tinnitus (Contribution 25), and the impact of emerging environmental factors, such as intensive video gaming [12] (Contribution 26).

The collected publications clearly indicate that the future of research on bruxism and TMD will rely on the integration of advanced diagnostic methods, identification of biopsychosocial determinants, and the development of personalised therapeutic strategies. This is particularly relevant in view of projections suggesting a further increase in the global prevalence of TMD by 2050 [13] (Contribution 27). This Special Issue provides an up-to-date summary of current knowledge while also outlining future directions for research into these complex disorders.

## Future Directions and Clinical Implications

Future research into bruxism and TMD should focus on further integrating the biopsychosocial approach through the use of advanced diagnostic methods and the development of therapies tailored to individual patient needs. Particular importance will be placed on developing more precise tools for assessing the risk of chronic pain development, identifying biomarkers, and gaining a better understanding of the interactions between genetic factors, sleep, stress, stomatognathic system function, and lifestyle. In clinical practice, it will be necessary to further strengthen interdisciplinary cooperation involving dentists, physiotherapists, doctors from other medical specialties, and mental health professionals. The increasing use of technologies such as surface electromyography, ultrasonography, elastography, magnetic resonance imaging, and digital tools for monitoring muscle activity may contribute to earlier detection of disorders and more effective treatment planning. At the same time, future therapeutic strategies should address not only symptom reduction but also the modification of risk factors and the improvement of patients’ long-term quality of life.
